# Expanding the genetic toolset: using serine recombinases to integrate riboregulatory elements into industrially relevant microbial chassis

**DOI:** 10.1093/jimb/kuag015

**Published:** 2026-06-02

**Authors:** Katherine L Wozniak, Austin L Carroll, Steffi A Davison, Ramesh K Jha, Adam M Guss, Scott P Hennelly, Taraka Dale, Chris M Yeager

**Affiliations:** Chemistry Division, Los Alamos National Laboratory, Los Alamos, United States; Agile Biofoundry, Emeryville, United States; Agile Biofoundry, Emeryville, United States; Biosciences Division, Oak Ridge National Laboratory, Oak Ridge, United States; Agile Biofoundry, Emeryville, United States; Biosciences Division, Los Alamos National Laboratory, Los Alamos, United States; Agile Biofoundry, Emeryville, United States; Biosciences Division, Los Alamos National Laboratory, Los Alamos, United States; Agile Biofoundry, Emeryville, United States; Biosciences Division, Oak Ridge National Laboratory, Oak Ridge, United States; Agile Biofoundry, Emeryville, United States; Biosciences Division, Los Alamos National Laboratory, Los Alamos, United States; Agile Biofoundry, Emeryville, United States; Biosciences Division, Los Alamos National Laboratory, Los Alamos, United States; Agile Biofoundry, Emeryville, United States; Biosciences Division, Los Alamos National Laboratory, Los Alamos, United States

**Keywords:** riboregulator, serine recombinase, *Pseudomonas putida*, *Corynebacterium glutamicum*, *Cupriavidus necator*

## Abstract

To realize the full potential of biomanufacturing, the breadth of industrial microbes used to consume diverse feedstock and generate bioproducts needs to expand. As such, portable tools are required that can be used by multiple hosts for straightforward genomic manipulation and precise gene expression. Here, we demonstrate the co-utilization of two synthetic biology tools to achieve these goals: *cis*-repressors (CRs) and serine recombinase-assisted genome engineering (SAGE). CRs are small, noncoding RNAs that are placed upstream of the target gene to modulate bacterial translation rates at varying, discrete levels. SAGE uses site-specific serine recombinases to catalyze highly efficient, unidirectional insertion of DNA into the chromosome of diverse organisms. We used SAGE to integrate a suite of CRs into the industrially relevant hosts *Pseudomonas putida, Corynebacterium glutamicum*, and *Cupriavidus necator*. Using a fluorescent reporter as a readout of CR functionality, we found that CR performance across these backgrounds was similar—providing a range of translational repression up to 100-fold. Overall, these results demonstrate the high portability of CRs across bacterial genetic backgrounds, which ideally can be used in future microbial engineering efforts pertinent to biomanufacturing.

**One-Sentence Summary**: Fine tuning translation across phylogenetically diverse microorganisms using riboregulators and serine recombinase-assisted genome engineering.

## Graphical Abstract

**Graphical Abstract:**
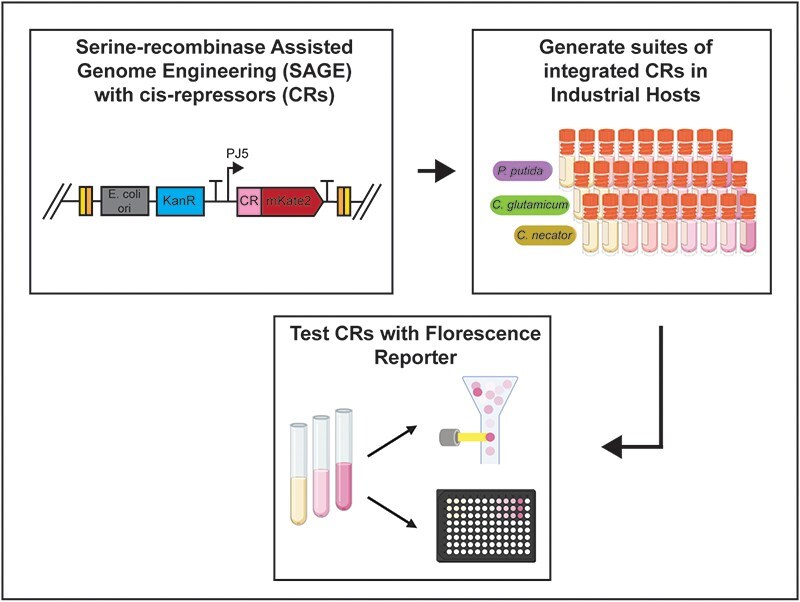

## Introduction

Bio-based products are critical for global solutions in energy security and climate change and are a growing fraction of the commodity market (Rinke Dias de Souza et al., 2024; Sohn et al., 2020). However, only a handful of host microorganisms have been sufficiently "domesticated" for use as efficient bio-production platforms (Calero & Nikel, 2019; Chaudhary et al., 2024; Xu et al., 2020). To realize biomanufacturing’s full potential, we must expand the scope of domesticated hosts, particularly those more suited to industrial conditions and with the metabolic versatility to broaden the types of feedstocks used and chemicals produced (Bales et al., 2024; Chan et al., 2025; Poppeliers et al., 2025; Riley & Guss, 2021; Yeager et al., 2025).

One of the challenges in developing nontraditional hosts as production chassis has been the difficulty of porting tools to modulate translation across phylogenetically diverse microorganisms (Bervoets & Charlier, 2019; Isaacs et al., 2004; Jin et al., 2017; Kent & Dixon, 2020; Liu et al., 2023; Sakai et al., 2014). Recently, we developed a suite of small, *cis*-acting, noncoding RNAs, called *riboregulators*, as tools to inhibit translation at varying, discrete levels (Krishnamurthy et al., 2015; Pandey et al., 2022), which use similar approaches to the synthetic riboregulators and programmable RNA switches described by others (Green et al., 2014; Kim et al., 2019; Mutalik et al., 2012). RNA-based technologies such as these offer more flexible regulation compared to promoter control, and they do not require additional cofactors to function (Groher & Suess, 2014; Jung et al., 2021). Our riboregulators, or *cis*-repressors (CRs), are placed upstream of a target gene, where they form an RNA hairpin structure that partially occludes the ribosomal binding site (RBS). The stability of the CR–RBS hairpin, and its corresponding strength as a repressor of gene expression, can be adjusted by altering the sequence of the CR (Krishnamurthy et al., 2015; Pandey et al., 2022). Thus far, we have tested our CRs in 2 members of the class Gammaproteobacteria, *Escherichia coli* and *Pseudomonas putida*, where we highlighted this tool’s potentially agnostic nature with regards to the sequence location (plasmid expressed vs. integrated into the genome), upstream promoter, and reporter gene; furthermore, we showed that CRs could effectively modulate pathway flux in *P. putida* (Pandey et al., 2022).

**Figure 1:**
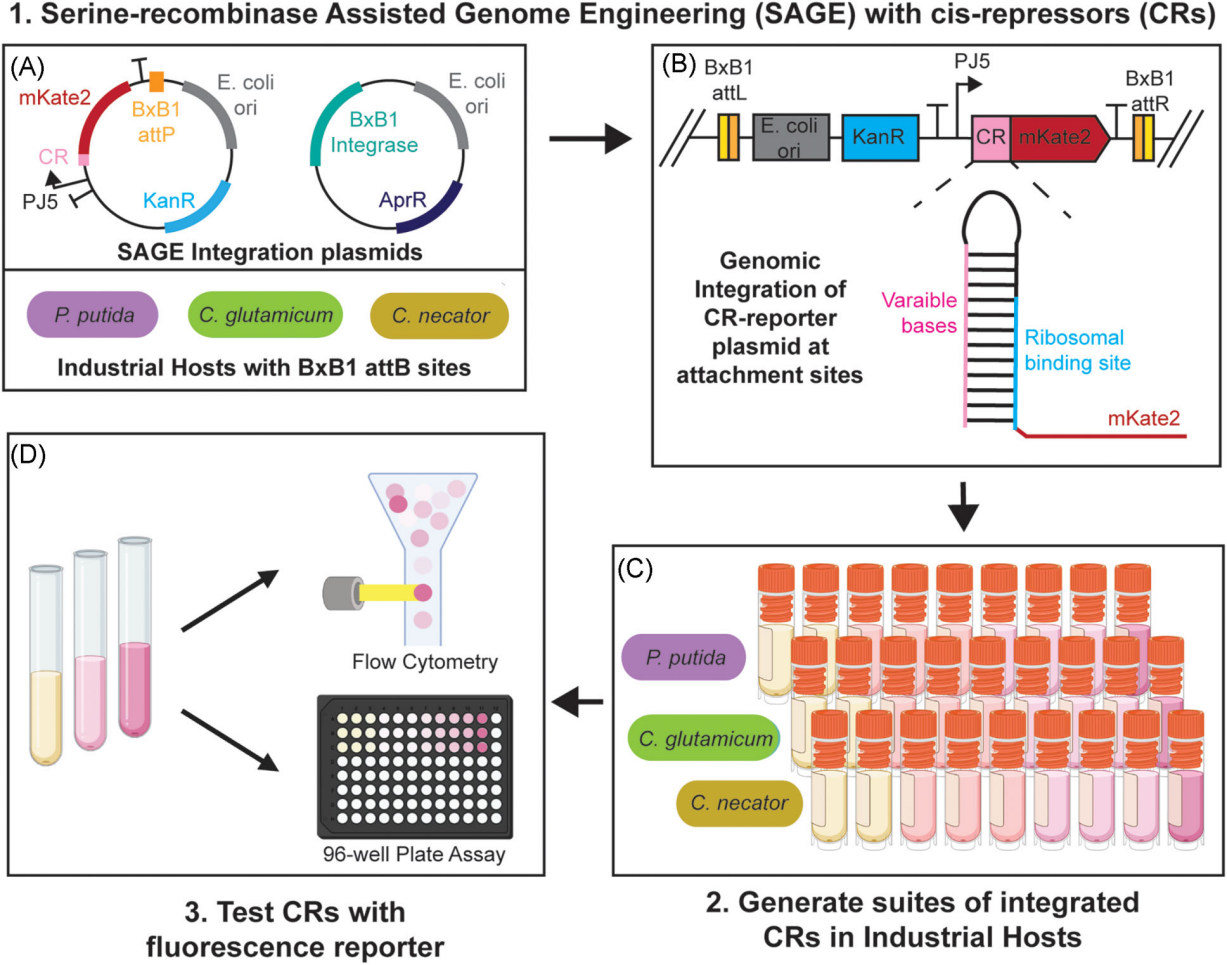
Workflow for using SAGE to efficiently integrate and test CRs in multiple industrial bacterial hosts. (A) Two plasmids were used for integration: (1) the CR-mKate2 plasmid with a BxB1 *attP* site and (2) the BxB1 recombinase plasmid. Neither of these plasmids can replicate in the target organisms. (B) Each CR-mKate2 plasmid was integrated into host genomes fitted with an *attB* site, thereby generating *attL* and *attR* sites. (C) Strains containing different CR sequences upstream of reporter genes were isolated and verified by sequencing to yield a library of strains containing 8 different CR-mKate2 constructs integrated in the same site in the genome. (D) Constitutive CR activity was tested using the mKate2 fluorescence reporter in flow cytometry assays to measure gene expression at the single-cell level and representing the fluorescence value as a geometric median and by evaluating cultures in 96-well plates to measure gene expression at the clonal population level. Image created with BioRender.com.

Another challenge in domesticating nontraditional hosts is that traditional genomic integration strategies are often time-consuming and have low portability across bacterial hosts (Kent & Dixon, 2020; Nora et al., 2019; Riley & Guss, 2021). To that end, we recently developed a system called serine recombinase-assisted genome engineering (SAGE) (Elmore et al., 2023). SAGE utilizes site-specific serine recombinases that catalyze unidirectional DNA recombination between 2 nonidentical recombination sites called *attB* and *attP* (Figure 1A and B) (Brown et al., 2011; Fogg et al., 2014; Merrick et al., 2018), and has been implemented in a wide range of microbial hosts (Elmore et al., 2023, 2017, 2025; Nava et al., 2023; Riley et al., 2023; Schmidt et al., 2023; Wilkes et al., 2024).

In this study, we present a more comprehensive analysis of the host-agnostic quality of our CR suite in *P. putida* and evaluate their performance in 2 additional phylogenetically distinct hosts. SAGE was used to integrate CRs into 3 up-and-coming industrial chassis, namely *Corynebacterium glutamicum* (Actinobacteria), *Cupriavidus necator* (Betaproteobacteria), and *P. putida* (Gammaproteobacteria) (Figure 1), to quickly test the efficacy and reproducibility of CR performance. Given that these diverse hosts can utilize the same promoter (*P*_J5_), fluorescent reporter (mKate2), and are sensitive to the same antibiotic (kanamycin) (Elmore et al., 2017; Nakamura et al., 2025; Schmidt et al., 2023), we made one set of plasmids to integrate our suite of CRs into all 3 species. We evaluated the performance of CRs both at a single cell level using flow cytometry and in 96-well format using a plate reader for the cumulative response of the population.

## Materials and methods

KOD Xtreme Hot Start DNA Polymerase (Sigma) was used for all polymerase chain reactions (PCR). gBlocks and primers were synthesized by Integrated DNA Technologies. Restriction enzymes, NEBuilder HiFi DNA Assembly Master Mix, and high-efficiency 5α competent *E. coli* cells (C2987H) were purchased from New England Biolabs Inc. (NEB). Digestions, Gibson assemblies, and transformations were performed according to manufacturer (NEB) instructions. Transformants were selected on Luria–Bertani (LB) agar (Fisher) plates with 50 µg/mL kanamycin (Sigma), 50 apramycin (Alfa Aesar), or 20 µg/mL chloramphenicol (Fisher). Sanger sequencing was used to confirm sequences of all plasmids (Azenta Life Sciences). For a complete list of all primers and plasmids used, see Supplementary Tables S1 and S2.

*Pseudomonas putida* was grown in LB medium or M9 minimal medium (M9), with or without 50 µg/mL kanamycin. M9 medium consisted of 6.78 g/L Na_2_HPO_4_, 3 g/L KH_2_PO_4_, 0.5 g/L NaCl, 1 g/L NH_4_Cl, 2 mM MgSO_4_, 100 μM CaCl_2_, 18 μM FeSO_4_, and 30 mM glucose. *Corynebacterium glutamicum* was grown in brain heart infusion-supplemented (BHIS) medium (18.5 g/L BHI, 91 g/L sorbitol; pH 7.2) ± 25 µg/mL kanamycin or 10 µg/mL chloramphenicol. *Cupriavidus necator* was grown in LB medium ± 200 µg/mL kanamycin.

*Pseudomonas putida* electrocompetent cells were prepared as described by Choi et al. (2006). Transformations were performed as described by Johnson and Beckham (2015) using 300–400 ng CR-mKate2 plasmid and 200 ng recombinase plasmid. Cells were recovered in SOC medium and plated on LB agar with 50 µg/mL kanamycin.

*Corynebacterium glutamicum* electrocompetent cell preparation and transformations were performed as described by Ruan et al. (2015), using 200–300 ng CR-mKate2 plasmid and 200 ng recombinase plasmid, or 100 ng CR-superfolder green fluorescent protein (sfGFP) plasmid. Cells were recovered in BHI medium (37 g/L BHI, 10 g/L (NH_4_)_2_SO_4_, 0.2 g/L K_2_HPO_4_, 0.3 g/L NaH_2_PO_4_, and 0.5 g/L MgSO_4_; pH 7.2) and plated on LBHIS agar (5 g/L tryptone, 5 g/L NaCl, 2.5 g/L yeast extract, 18.5 g/L BHI, 91 g/L sorbitol, 18 g/L agar; pH 7.2) with 25 µg/mL kanamycin or 10 µg/mL chloramphenicol.

To prepare *Cupriavidus necator* cells for transformation, 25 mL of overnight culture was centrifuged (3,000 × *g*, 5 min), and the resulting cell pellet was gently suspended in 50 mM CaCl_2_ (25 mL) and incubated for 15 min at room temperature. The cells were then pelleted by centrifugation (3,000 × *g*, 10 min) and gently resuspended in 25 mL 10% glycerol; this wash step was repeated 2 additional times. Next, the cells were centrifuged (3,000 × *g*, 10 min), and the pellet was suspended in 0.5 mL 10% glycerol to yield electro-competent cells for transformation or storage at −80 °C. For transformation, 50 µL of competent cells was mixed with 200–300 ng of the CR-mKate2 plasmid and 300 ng recombinase plasmid at room temperature. The mixture was then loaded into a 0.1 mm cuvette and electroporated using a Gene Pulser Xcell (Bio-Rad, Hercules, CA) with the following settings: exponential waveform, 1.6 kV, 25 µF, 200 ohms. The resulting time constant for the transformation was typically > 4.0. Cells were recovered in SOC medium containing 20 mM fructose (0.5 mL) by shaking at 30 °C for 2–3 h, plated on LB agar with 200 µg/mL kanamycin and incubated overnight at 30 °C.

Correct integration of DNA for all hosts was evaluated using colony PCR with primers targeting flanking regions for the resulting *attL* and *attR* attachment sites after recombination between the *attB* and *attP* (Figure 1B). Integrations were confirmed using Sanger sequencing (Azenta Life Sciences). For a complete list of all strains used, see Supplementary Table S3.

### Fluorescence assay using flow cytometry and plate reader

Glycerol stocks of each strain were streaked on agar plates containing the appropriate rich medium and selective antibiotic and incubated at 30 °C overnight. Single colonies were used to inoculate 5 mL of the appropriate rich medium lacking antibiotic (except for *Corynebacterium glutamicum* plasmid expression with 10 µg/mL chloramphenicol) and incubated at 30 °C with shaking (250 rpm) overnight to obtain primary seed cultures.

Primary seed cultures were then used to inoculate select medium types for growth experiments (5 mL; rich or minimal medium without antibiotics, except for *Corynebacterium glutamicum* plasmid expression with 10 µg/mL chloramphenicol) to an optical density of 0.2 at 600 nm (OD_600_) as measured with a Tecan Infinite M200 plate reader. These experimental cultures were grown at 30 °C with shaking (250 rpm) until mid-log phase was reached (OD 0.5–1.0; 2–14 h depending on strain and medium), at which time a sub-sample was removed for single cell fluorescence analysis (mKate2: 561 nm laser and 610/20 nm filter; sfGFP: 488 nm laser and 530/30 nm filter) using a BD FACSAria III flow cytometer (BD Biosciences). Given size differences among the hosts, parameter and threshold setting on the flow cytometer varied. For each sample, 100,000 events were measured at a rate of 6,000–10,000 events per second.

Sub-samples (150 µL) collected from the experimental cultures were also added to individual wells of a 96-well, black-walled, flat clear-bottom microplate (Corning). OD_600_ and fluorescence (mKate2: excitation = 588 nm, emission = 633 nm, gain = 245; sfGFP: excitation = 485 nm, emission = 530 nm) were measured using a Tecan Infinite M200 plate reader. Fluorescence intensity was normalized to OD_600_.

## Results and discussion

We used the SAGE system to integrate the CR suite into multiple industrial hosts (Figure 1). A series of plasmids were constructed with CRs upstream of the fluorescent reporter mKate2; these plasmids also contained a common promoter (*P*_J5_), the kanamycin resistance (KanR) marker, and a BxB1 *attP* site (Figure 1A and Supplementary Table S2). Each CR–mKate2 plasmid was co-transformed with a BxB1 recombinase expression plasmid (Elmore et al., 2023), such that site-specific integration occurred using the CR plasmid-based *attP* site into the engineered genomic *attB* sites in *P. putida, Corynebacterium glutamicum*, and *Cupriavidus necator*. Each transformation yielded 1,000s of transformants; a handful of those were sequenced, confirming >95% had correct integrations.

**Figure 2:**
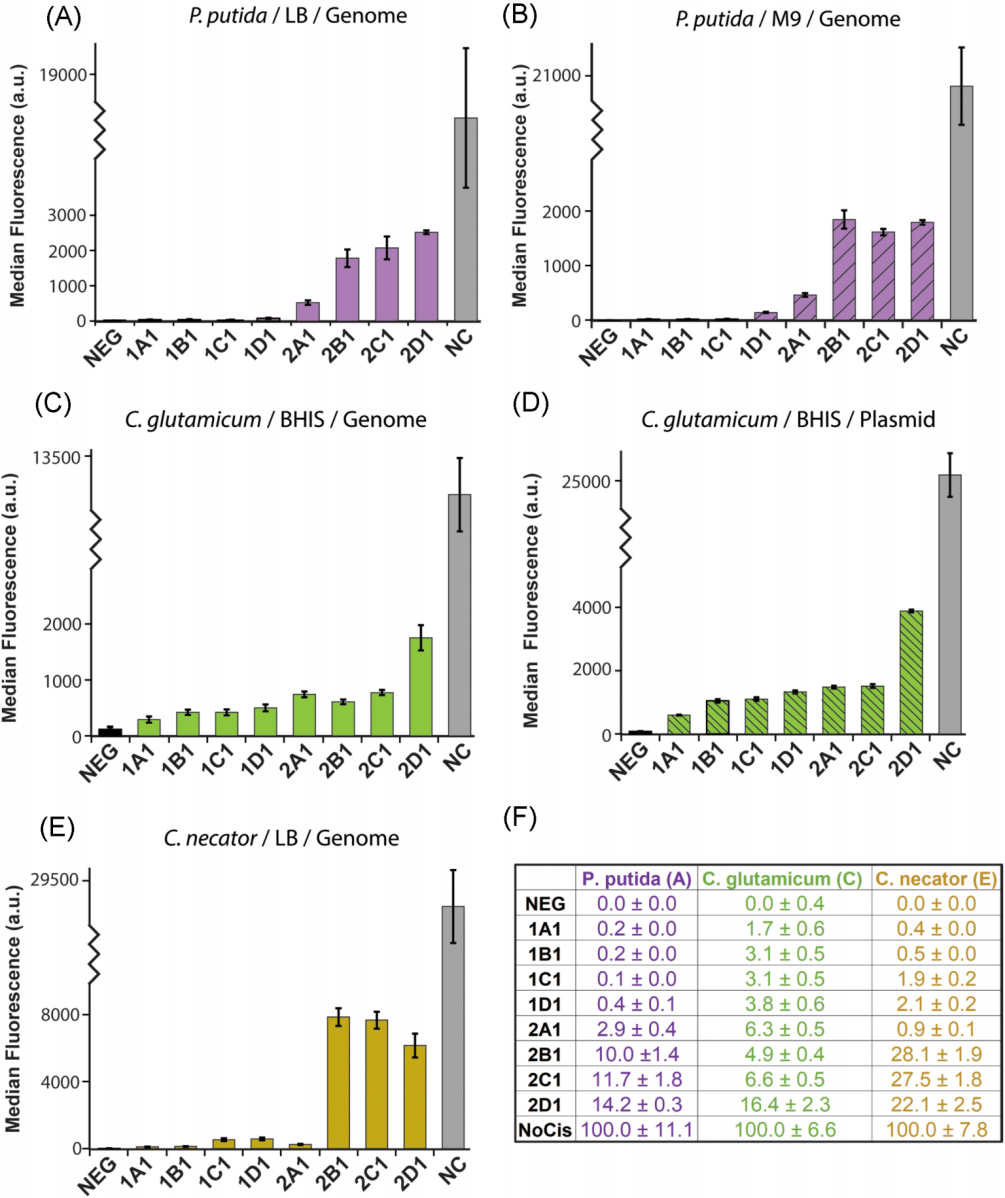
Expression profiles of CRs in industrially relevant hosts measured by flow cytometry. Median fluorescence profiles measured on the Aria flow cytometer for (A) a suite of CRs inserted into the *P. putida* genome when grown in LB rich medium, (B) the same CRs in the *P. putida* genome when grown in M9 minimal medium, (C) the same CRs in the *Corynebacterium glutamicum* genome when grown in BHIS medium, (D) the same CRs (minus 2B1) with the sfGFP reporter on plasmids in *Corynebacterium glutamicum* when grown in BHIS medium (with 10 µg/mL chloramphenicol), and (E) the same CRs in the *Cupriavidus necator* genome when grown in LB medium. Each experiment included a negative control lacking any reporter (NEG) and a positive control with an unrepressed reporter (NoCis, NC). Histograms of flow cytometry results can be found in Supplemental Figures 1–5. (F) Normalized (against the NC positive control) median fluorescence values for genome integrated CRs in *P. putida, Corynebacterium glutamicum*, and *Cupriavidus necator* cultures grown in rich media. Data show 3 biological replicates with standard deviations.

**Figure 3:**
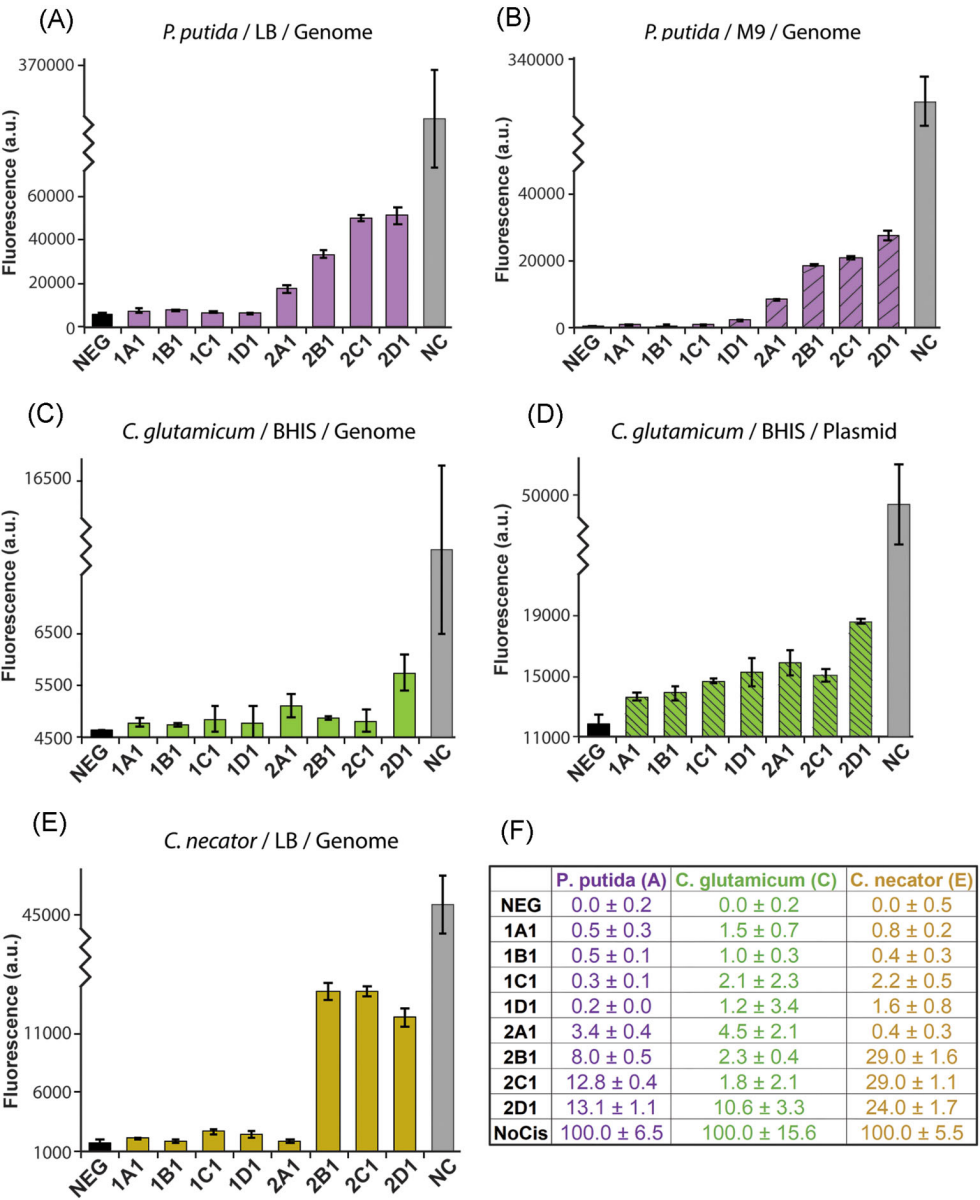
Expression profiles of CRs in industrial hosts measured by plate reader. Mean fluorescence profiles measured on the Tecan plate reader for (A) a suite of CRs inserted into the *P. putida* genome when grown in LB rich medium, (B) the same CRs in the *P. putida* genome when grown in M9 minimal medium, (C) the same CRs in the *Corynebacterium glutamicum* genome when grown in BHIS medium, (D) the same CRs (minus 2B1) with the sfGFP reporter on plasmids in *Corynebacterium glutamicum* when grown in BHIS medium (with 10 µg/mL chloramphenicol), and (E) the same CRs in the *Cupriavidus necator* genome when grown in LB medium. Each experiment included a negative control lacking any reporter (NEG) and a positive control with an unrepressed reporter (NoCis, NC). (F) Normalized (against the NC positive control) mean fluorescence values for genome-integrated CRs in *P. putida, Corynebacterium glutamicum*, and *Cupriavidus necator* cultures grown in rich media. Data show 3 biological replicates with standard deviations.

Fluorescence reporter intensity was used as a measure of CR performance across the different hosts, which was evaluated at the single cell level using flow cytometry and at the population level in 96-well format using a plate reader (Figure 1D; Supplementary Tables 4 and 5). To get optimal resolution on the flow cytometer, the parameters used to measure each strain were adjusted, thus the raw fluorescence values cannot be directly compared (Figure 2); however, the respective normalized median fluorescence values can be compared (Figure 2F). The same parameters were used to measure cumulative fluorescence of the population on the plate reader; thus, comparative inferences can be made using these data (Figure 3). We sought to collect both types of measurements to demonstrate multiple methods for testing these systems.

We tested if CR performance in *P. putida* was agnostic to the upstream promoter, reporter, and genome location by comparing CR-dependent expression levels in this study (*P*_j5_ promoter, mKate2 reporter, PP_4741 locus) with those previously obtained (*P*_tac_ promoter, sfGFP reporter, PP_2684-PP_2685 locus) (Pandey et al., 2022). Interestingly, the range of fluorescence values observed in this study was less than those observed in Pandey et al. (2022), potentially due to differences in levels of transcriptional activity across genome integration sites and difference in promoters. Yet the rank order of translation inhibition by each CR was generally similar in both studies, which also mimicked the *E. coli* results from Pandey et al. (Figures 2A and 3A). This is the first time that CRs 1B1, 1C1, 1D1, and 2A1 were evaluated in *P. putida*. Between these new CRs, there is a 17-fold range in translation levels (Figure 2E), thus demonstrating additional precision tools for *P. putida* engineering.

Using the entire suite of 8 CRs in *P. putida*, we found an ~100-fold range in gene expression, as determined by flow cytometry (Figure 2A and F). CR-1A1 exhibited one of the lowest fluorescence intensities (0.2 ± 0.0%), which was only slightly higher than the negative control strain that lacked mKate2 altogether (NEG). The strain containing the medium strength CR, 2A1, demonstrated moderate mKate2 fluorescence (2.9 ± 0.4%), while ones with the weakest CR (2D1) exhibited the highest mKate2 signal of any CR (14.2 ± 0.3 %).

To investigate if CR expression is influenced by growth conditions, we also assayed CR performance in *P. putida* transformants grown in M9 minimal medium (Figures 2B and 3B). The rank order of fluorescence intensity was similar whether cells were grown in LB or M9 minimal medium (e.g., 1A1 demonstrated weak mKate2 expression, 2A1 moderate expression, and 2D1 the highest CR expression) but differed in the overall extent of inhibition. For example, the most highly expressed CR (2D1) exhibited 60% higher fluorescence in rich medium compared to minimal medium (14.2 ± 0.3% vs. 8.7 ± 0.2%, respectively).

Next, we tested the cross-species portability of CRs by using SAGE to insert them into the *Corynebacterium glutamicum* genome. mKate2 expression in the NC control was higher in *P. putida* than *Corynebacterium glutamicum*, suggesting that the *P*_j5_ promoter is stronger in *P. putida* (Figure 3A and C). Due to the low fluorescence in *Corynebacterium glutamicum* cultures and replicate variability, most of the CR fluorescence profiles were indistinguishable using the plate reader (Figure 3C and F). However, a stepwise pattern of CR-dependent fluorescence intensity was observed using flow cytometry (Figure 2C and F). As observed in *P. putida* and *E. coli*, CR-1A1 and CR-2D1 exhibited the most and least repression, respectively (Figure 2C).

To confirm that the rank order of CR performance in *Corynebacterium glutamicum* was not dependent on genome integration site, upstream promoter, or target gene, we generated a suite of plasmid-based CRs using superfolder sfGFP as a reporter under control of the *P*_tac_ promoter (Figures 2D and 3D). In contrast to the *Corynebacterium glutamicum* cultures with genome-integrated CR-mKate2 constructs, the cultures with plasmid-borne CR-sfGFP constructs exhibited a stepwise inhibition pattern of fluorescence when measured using both the plate reader and flow cytometry. Comparing CR performance using the plasmid-based sfGFP reporter, CR-1A1 and CR-2D1 again exhibited the most and least repression, respectively. These results suggest that CR rank order performance in *Corynebacterium glutamicum* is largely agnostic toward adjacent genetic elements or whether the construct resides on a plasmid or is integrated into the genome.

As a final portability test, we integrated the CRs into the *Cupriavidus necator* genome using SAGE (Figures 2E and 3E). The general pattern of CR activity in *Cupriavidus necator* was slightly different than that observed in other hosts. Rather than exhibiting a stepwise pattern, the CRs in *Cupriavidus necator* could be grouped into 2 levels of inhibitors: potent (>97% inhibition) and moderate (70%–80% inhibition) (Figures 2F and 3F). Consistent with the other hosts tested, CR-1A1 and CR-2D1, respectively, fell into the most and one of the least repression categories, respectively.

Between the current study and our previous one (Pandey et al., 2022), we have demonstrated that CRs act similarly across 4 diverse bacterial backgrounds (*P. putida, Corynebacterium glutamicum, Cupriavidus necator*, and *E. coli)*, 3 promoters (*P*_j5_, *P*_tac_, and T7A1), and 4 effector genes (coding for mKate2, sfGFP, chloramphenicol acetyltransferase, and PPsA). Overall, the results demonstrate that similar suites of CRs can be rapidly screened, across different hosts and genomic integration sites, to identify those that yield the desired level of translational inhibition. While the rank-order of CRs was relatively consistent across hosts, we do note that differences were observed in the precise order and range of expression levels for CRs that perform similarly. When translating CRs into novel organisms, we recommend evaluating several CRs to identify a potent, moderate, and weak one for further use. While we initially evaluated 15 CRs in *E. coli* (Pandey et al., 2022), we reduced this number to 8 in this study to limit redundancy of similar CRs; given that we observed 3 discrete groups of CRs (low, moderate, and high inhibition), fewer than 8 CRs could likely be tested in other systems to obtain desired levels of translational tuning. For many cases, it is desirable to fine tune low levels of gene expression, thus the overabundance of CRs exhibiting potent or moderate inhibition may be operationally useful. Once CRs that yield differential levels of inhibition have been validated, they could be applied to optimize translation rates at key points in a desired pathway, as we previously demonstrated for muconic acid production in *P. putida* (Pandey et al., 2022). Furthermore, we verified here that SAGE system offers a quick and efficient means for testing genomically integrated synthetic tools. Future studies that apply the SAGE system with CRs to nonmodel hosts will expedite their domestication by providing a means for rapidly testing translational tuning.

## Supplementary Material

kuag015_Supplemental_File

## Data Availability

All data described in this manuscript are available upon request.
