# Supplementary material for: Expanding the genetic toolset: using serine recombinases to integrate riboregulatory elements into industrially relevant microbial chassis
Source: J Ind Microbiol Biotechnol. 2026 Jun 2;53:kuag015. doi: 10.1093/jimb/kuag015 (PMC13289732; doi:10.1093/jimb/kuag015)
Supplement: kuag015_Supplemental_File [file kuag015_supplemental_file.docx]

**Supplementary Material**

Supplemental Figures 1-5. Normalized (against the NC positive control) median fluorescence values for genome integrated CRs in *P. putida*, *C. glutamicum*, and *C. necator* cultures grown in rich media. Data show three biological replicates with standard deviations.

**
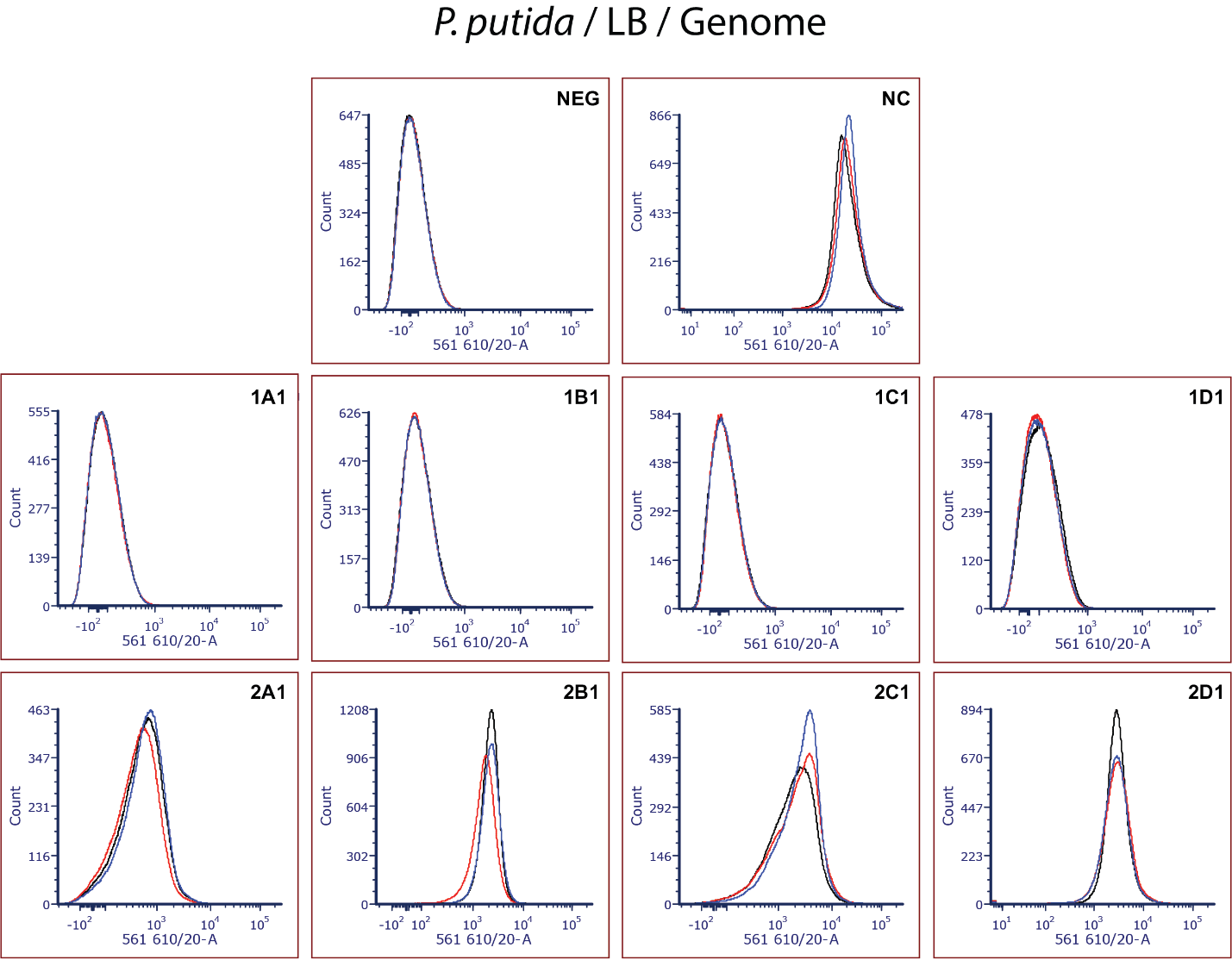
**

**Supplemental Figure 1.** Histogram of flow cytometry data for a suite of CRs inserted into the *P. putida* genome when grown in LB rich medium.


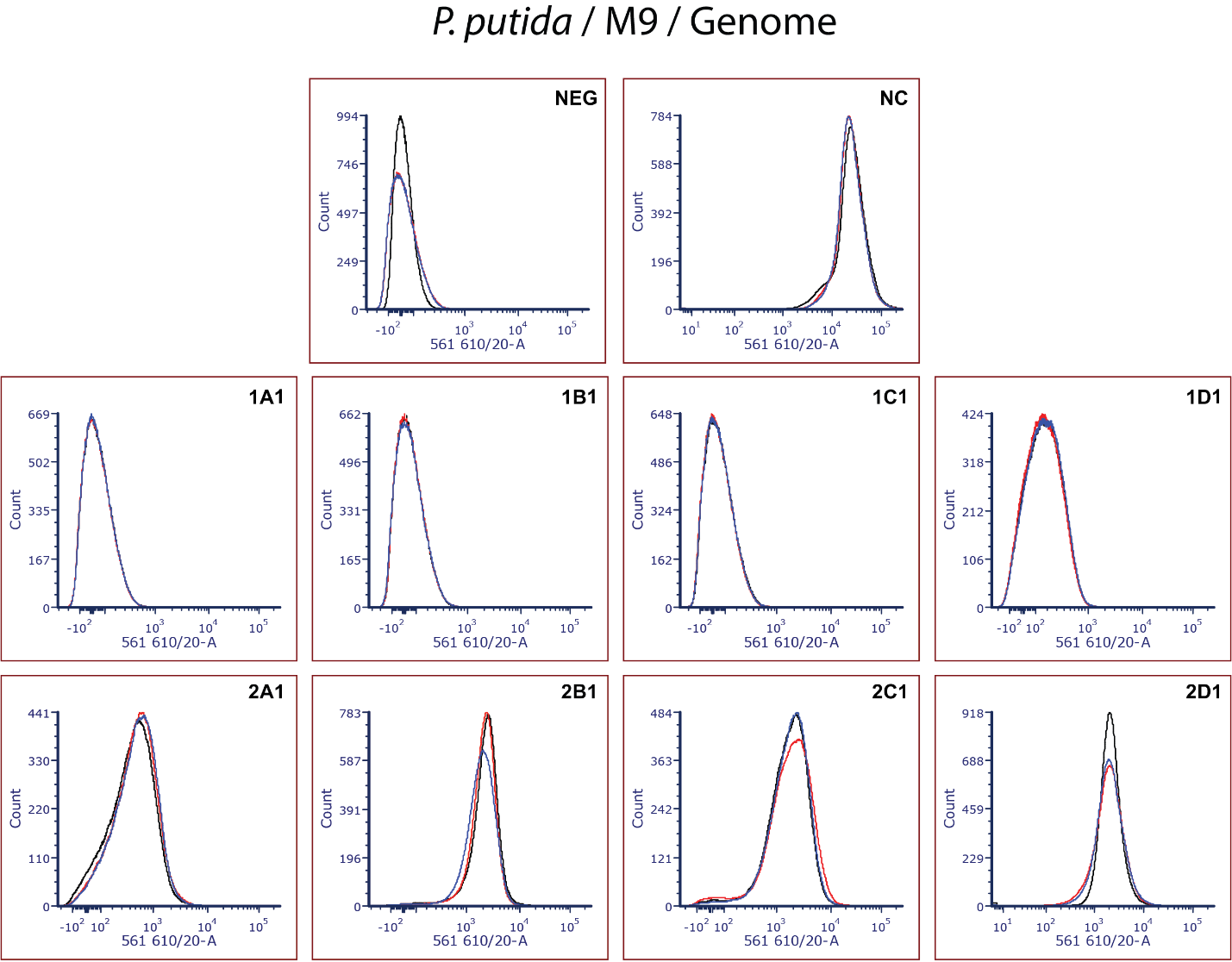


**Supplemental Figure 2.** Histogram of flow cytometry data for a suite of CRs inserted into *the P. putida* genome when grown in M9 minimal medium.

**
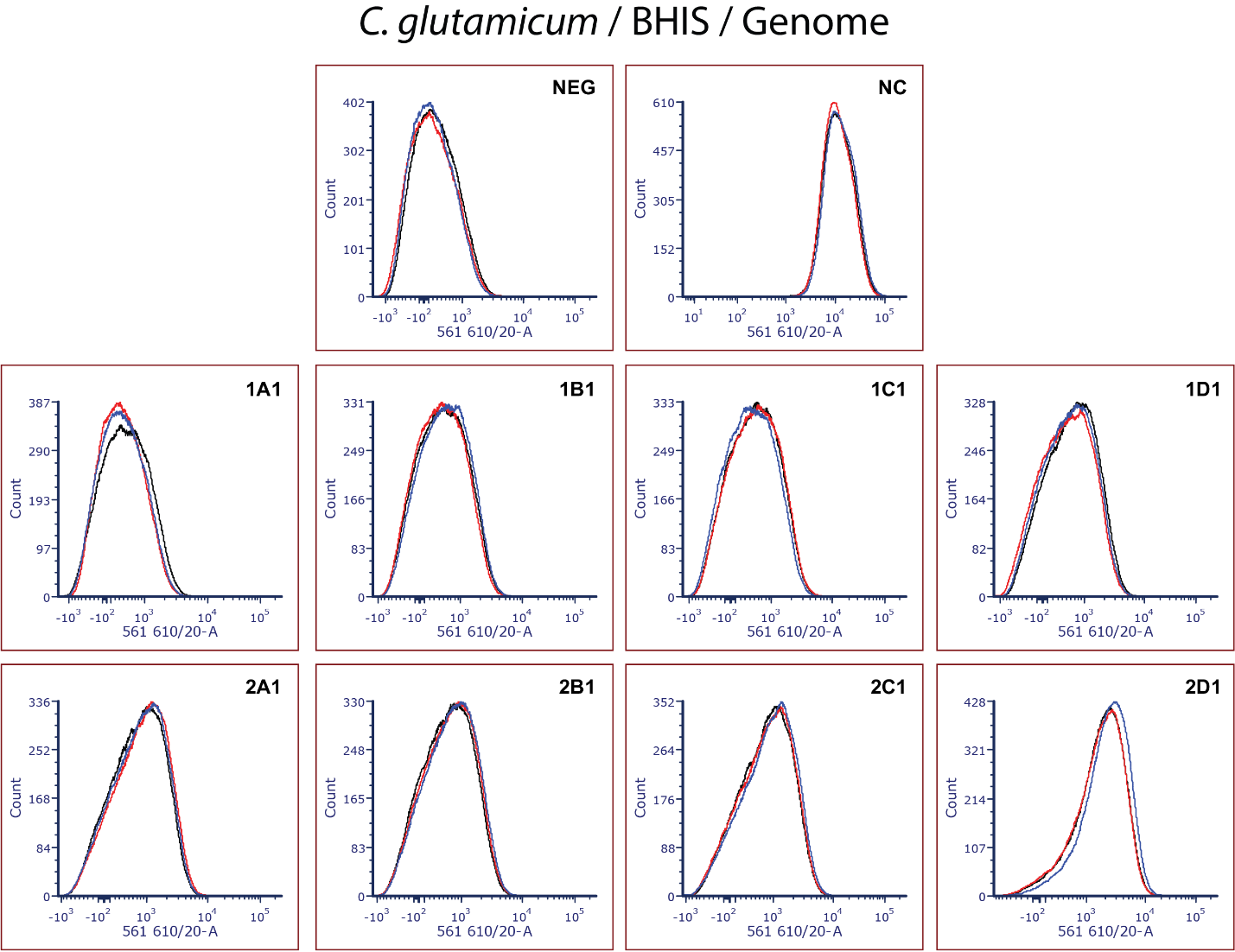
**

**Supplemental Figure 3.** Histogram of flow cytometry data for a suite of CRs inserted into the *C. glutamicum* genome when grown in BHIS medium.

**
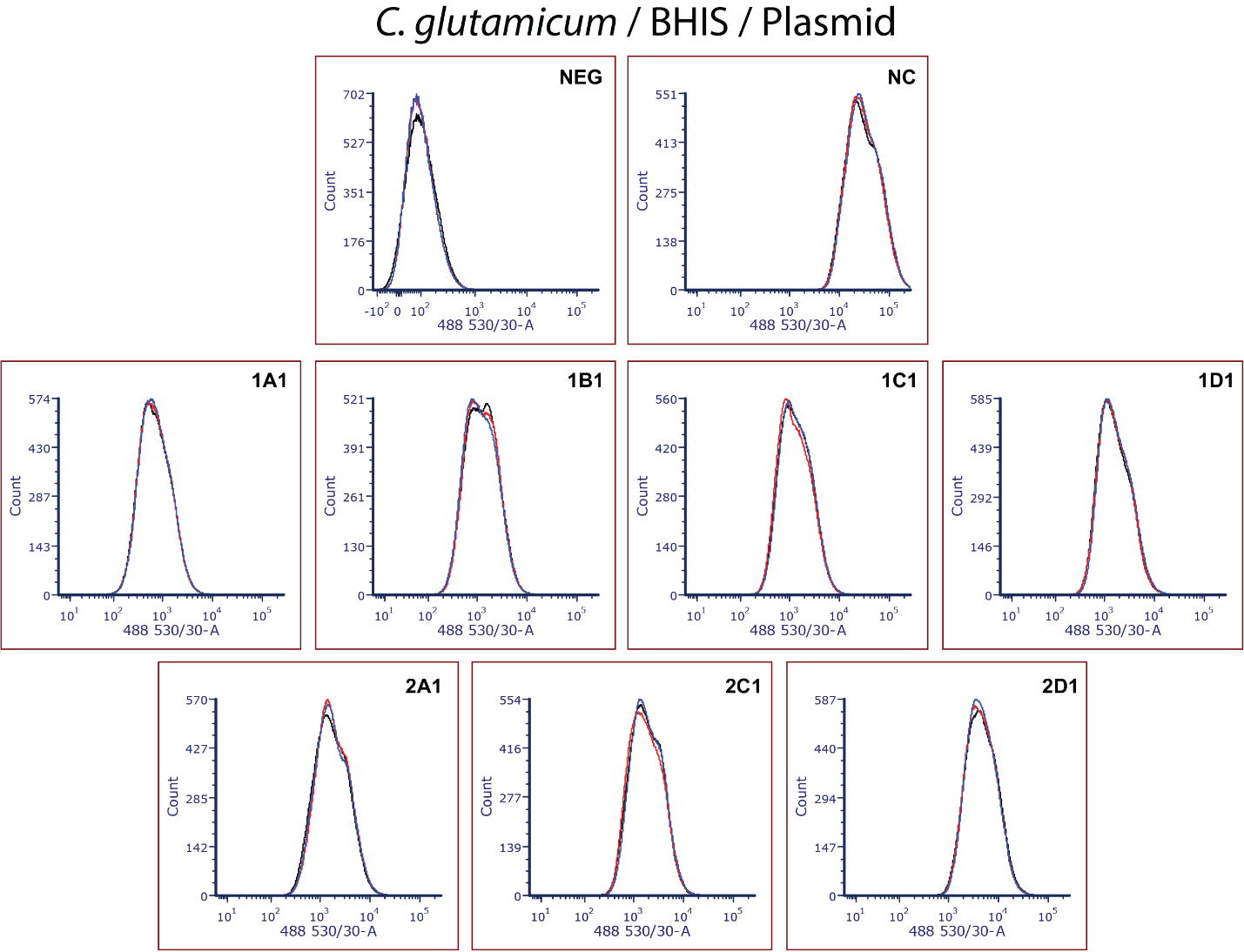
**

**Supplemental Figure 4.** Histogram of flow cytometry data for a suite of CRs with the sfGFP reporter on plasmids in *C. glutamicum* when grown in BHIS medium (with 10 µg/mL chloramphenicol).

**
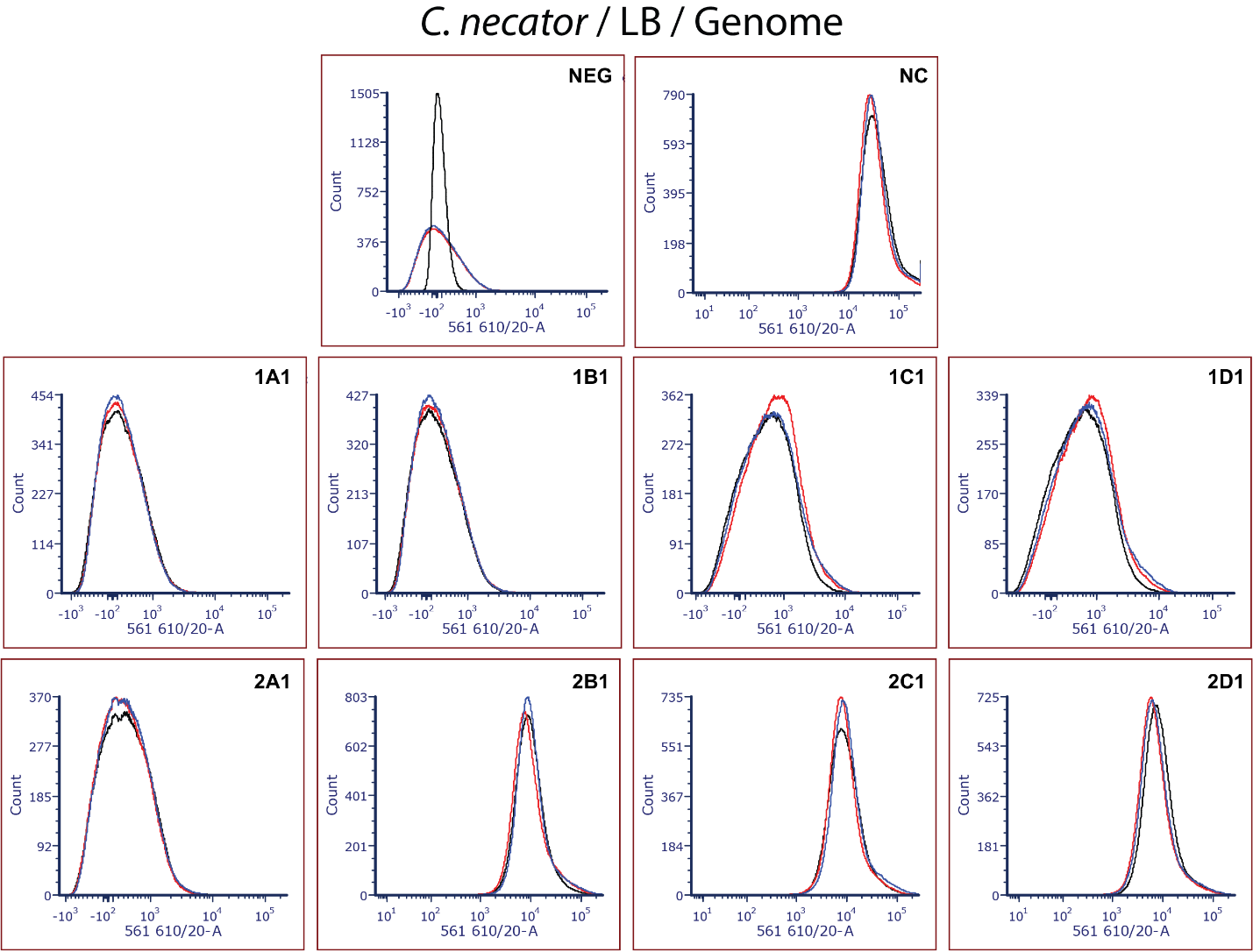
**

**Supplemental Figure 5.** Histogram of flow cytometry data for a suite of CRs inserted into the *C. necator* genome when grown in LB medium.

**Supplemental Table 1.** Primers and G-blocks

| **Primer Name** | **Primer Sequence (5’ to 3’)** | **Reference** |
| --- | --- | --- |
| PJ5-CRgb_mKate2_F | ggaattgtgagcggataacaattcttaagattacattttcttgtctcaaacaataagc | This study |
| PJ5-CRgb_mKate2_R | tgagttcgctgaccattcgaacctctccttctatcgacgc | This study |
| CR_mKate2_mK2_F | ggagaggttcgaatggtcagcgaactcattaaagagaacatgc | This study |
| CR_mKate2_mK2_R | catcctcatacgtggtgacgcgttcccacgtaaacccttctg | This study |
| pRJ_nocis_mKate2_F | ggaattgtgagcggataacaattcttaaggaaatcgtaatgcgtcgatagaag | This study |
| PJ5_CR-mKate2_screen_F | ctatggaggtcaggtatgattac | This study |
| PJ5_CR-mKate2_screen_R | cacattgtagatcaggcagc | This study |
| attL_screen_F | cttgtcgacgacggcggtc | This study |
| M13_57_R | gcggataacaatttcacacaggaaac | This study |
| attR_screen_R | gcccggatgatcctgacgacgg | This study |
| F_pRJ_Kpni_pTac_cis | cttggtctatagtggctaggtaccccctcaagtcaaaagcctc | This study |
| R_CR_pRJ_sfGFP | aaagttcttctcctttgctagccattcgaacctctccttctatcgacgcatt | This study |
| F_pRJ_screen_kpnI | tgaatcgaatttcggggctttaaagca | This study |
| F_NheI_CR | cattttcttgtctcaaacaataagctagc | This study |
| G-block 1B1 | CTATAGGATACTTACAGCCATCGAGAGGACGTCGGAATTGTGAGCGGATAACAATTCCAGAGGGAATAGGAATTGTGAGCGGATAACAATTTAAGGTTAGTGTGATTACATTTTCTTGTCTCAAACAATAAGCTAGCATTCAAGACTCCCCGTCTATCGGCGTGTGACGAGAAATCGTAATGCGTCGATAGAAGGAGAGGTTCGAATGAGCAAAGGAGAAGAACTTTTCAC | Pandey et al. (2022) |
| G-block 1C1 | CTATAGGATACTTACAGCCATCGAGAGGACGTCGGAATTGTGAGCGGATAACAATTCCAGAGGGAATAGGAATTGTGAGCGGATAACAATTTAAGGTTAGTGTGATTACATTTTCTTGTCTCAAACAATAAGCTAGCATTCGAGACTCCCCGTCTATCGGAGTGTGACGAGAAATCGTAATGCGTCGATAGAAGGAGAGGTTCGAATGAGCAAAGGAGAAGAACTTTTCAC | Pandey et al. (2022) |
| G-block 1D1 | CTATAGGATACTTACAGCCATCGAGAGGACGTCGGAATTGTGAGCGGATAACAATTCCAGAGGGAATAGGAATTGTGAGCGGATAACAATTTAAGGTTAGTGTGATTACATTTTCTTGTCTCAAACAATAAGCTAGCATTCGAGACTCCCCGTCTATAGGCGTGTGACGAGAAATCGTAATGCGTCGATAGAAGGAGAGGTTCGAATGAGCAAAGGAGAAGAACTTTTCAC | Pandey et al. (2022) |
| G-block 2A1 | CTATAGGATACTTACAGCCATCGAGAGGACGTCGGAATTGTGAGCGGATAACAATTCCAGAGGGAATAGGAATTGTGAGCGGATAACAATTTAAGGTTAGTGTGATTACATTTTCTTGTCTCAAACAATAAGCTAGCATTCAAGACTCTCCGTCTATCGGAGTATGACGAGAAATCGTAATGCGTCGATAGAAGGAGAGGTTCGAATGAGCAAAGGAGAAGAACTTTTCAC | Pandey et al. (2022) |
| G-block 2B1 | CTATAGGATACTTACAGCCATCGAGAGGACGTCGGAATTGTGAGCGGATAACAATTCCAGAGGGAATAGGAATTGTGAGCGGATAACAATTTAAGGTTAGTGTGATTACATTTTCTTGTCTCAAACAATAAGCTAGCATTCGAGCCTCCCCGTCTATAGGAGTGTGACGAGAAATCGTAATGCGTCGATAGAAGGAGAGGTTCGAATGAGCAAAGGAGAAGAACTTTTCAC | Pandey et al. (2022) |
| G-block 2C1 | CTATAGGATACTTACAGCCATCGAGAGGACGTCGGAATTGTGAGCGGATAACAATTCCAGAGGGAATAGGAATTGTGAGCGGATAACAATTTAAGGTTAGTGTGATTACATTTTCTTGTCTCAAACAATAAGCTAGCATTCGAGACTCCCCGTCTATAGGAGTGTGACGAGAAATCGTAATGCGTCGATAGAAGGAGAGGTTCGAATGAGCAAAGGAGAAGAACTTTTCAC | Pandey et al. (2022) |
| G-block 2D1 | CTATAGGATACTTACAGCCATCGAGAGGACGTCGGAATTGTGAGCGGATAACAATTCCAGAGGGAATAGGAATTGTGAGCGGATAACAATTTAAGGTTAGTGTGATTACATTTTCTTGTCTCAAACAATAAGCTAGCATTCGAGACTCTCCGTCTATAGGAGTGTGACGAGAAATCGTAATGCGTCGATAGAAGGAGAGGTTCGAATGAGCAAAGGAGAAGAACTTTTCAC | Pandey et al. (2022) |

**Supplemental Table 2:** Plasmids

| **Plasmid Name** | **Genotype** | **Reference** |
| --- | --- | --- |
| pGW31 | BxB1 integrase plasmid including Apramycin Resistance (AprR) | Elmore et al. (2023) |
| pALC317 | Backbone for gene replacement including PJ5, mKate2, and KanR sequences | This study |
| pKW059 | PJ5 promoter, 1A1 CR, mKate2, and KanR | This study |
| pKW060 | PJ5 promoter, 1B1 CR, mKate2, and KanR | This study |
| pKW061 | PJ5 promoter, 1C1 CR, mKate2, and KanR | This study |
| pKW062 | PJ5 promoter, 1D1 CR, mKate2, and KanR | This study |
| pKW063 | PJ5 promoter, 2A1 CR, mKate2, and KanR | This study |
| pKW064 | PJ5 promoter, 2B1 CR, mKate2, and KanR | This study |
| pKW065 | PJ5 promoter, 2C1 CR, mKate2, and KanR | This study |
| pKW066 | PJ5 promoter, 2D1 CR, mKate2, and KanR | This study |
| pKW067 | PJ5 promoter, no CR, mKate2, and KanR | This study |
| pRJ2010 | Backbone for gene replacement including sfGFP and chloramphenicol resistance (CmR) sequences | Velasquez et al. (2024) |
| pBTL-2_ptac_1A1cr_sfGFP | P*_tac_* promoter, 1A1 CR, and sfGFP sequence cloned | Pandey et al. (2022) |
| pBTL02_ptac_NoCis_sfGFP | P*_tac_* promoter, no CR, and sfGFP sequence cloned | Pandey et al. (2022) |
| pKW001 | P*_tac_* promoter, 1A1 CR, sfGFP, and CmR | This study |
| pKW006 | P*_tac_* promoter, 1B1 CR, sfGFP, and CmR | This study |
| pKW011 | P*_tac_* promoter, 1C1 CR, sfGFP, and CmR | This study |
| pKW010 | P*_tac_* promoter, 1D1 CR, sfGFP, and CmR | This study |
| pKW005 | P*_tac_* promoter, 2A1 CR, sfGFP, and CmR | This study |
| pKW004 | P*_tac_* promoter, 2C1 CR, sfGFP, and CmR | This study |
| pKW009 | P*_tac_* promoter, 2D1 CR, sfGFP, and CmR | This study |
| pKW007 | ptac promoter, no CR, sfGFP, and CmR | This study |

**Supplemental Table 3:** Strains

| **Strain Name** | **Genotype** | **Reference** |
| --- | --- | --- |
| AG4063 | *P. putida* with *attB* site inserted into the chromosome (BxB1 landing pad) | This paper, modified from Elmore et al. 2023 |
| KW068P | *P. putida* AG4063 *PJ5:1A1:mKate2* | This study |
| KW069P | *P. putida* AG4063 *PJ5:1B1:mKate2* | This study |
| KW070P | *P. putida* AG4063 *PJ5:1C1:mKate2* | This study |
| KW071P | *P. putida* AG4063 *PJ5:1D1:mKate2* | This study |
| KW072P | *P. putida* AG4063 *PJ5:2A1:mKate2* | This study |
| KW073P | *P. putida* AG4063 *PJ5:2B1:mKate2* | This study |
| KW074P | *P. putida* AG4063 *PJ5:2C1:mKate2* | This study |
| KW075P | *P. putida* AG4063 *PJ5:2D1:mKate2* | This study |
| KW076P | *P. putida* AG4063 *PJ5:NoCis:mKate2* | This study |
| AG6212 | *C. glutamicum* with *attB* site inserted into the chromosome (BxB1 landing pad) | This study |
| KW068C | *C. glutamicum AG6212 PJ5:1A1:mKate2* | This study |
| KW069C | *C. glutamicum AG6212 PJ5:1B1:mKate2* | This study |
| KW070C | *C. glutamicum AG6212 PJ5:1C1:mKate2* | This study |
| KW071C | *C. glutamicum AG6212 PJ5:1D1:mKate2* | This study |
| KW072C | *C. glutamicum AG6212 PJ5:2A1:mKate2* | This study |
| KW073C | *C. glutamicum AG6212 PJ5:2B1:mKate2* | This study |
| KW074C | *C. glutamicum AG6212 PJ5:2C1:mKate2* | This study |
| KW075C | *C. glutamicum AG6212 PJ5:2D1:mKate2* | This study |
| KW076C | *C. glutamicum AG6212 PJ5:NoCis:mKate2* | This study |
| ATCC_13032 | *C. glutamicum* ATCC #13032 | ATCC #13032 |
| KW001C | *C. glutamicum ATCC #13032* P*_tac_:1A1:sfGFP* | This study |
| KW006C | *C. glutamicum ATCC #13032* P*_tac_:1B1:sfGFP* | This study |
| KW011C | *C. glutamicum ATCC #13032* P*_tac_:1C1:sfGFP* | This study |
| KW010C | *C. glutamicum ATCC #13032* P*_tac_:1D1:sfGFP* | This study |
| KW005C | *C. glutamicum ATCC #13032* P*_tac_:2A1:sfGFP* | This study |
| KW004C | *C. glutamicum ATCC #13032* P*_tac_:2C1:sfGFP* | This study |
| KW009C | *C. glutamicum ATCC #13032* P*_tac_:2D1:sfGFP* | This study |
| KW007C | *C. glutamicum ATCC #13032* P*_tac_:NoCis:sfGFP* | This study |
| AG5284 | *C. necator* with *attB* site inserted into the chromosome (BxB1 landing pad) | This study |
| KW068N | *C. necator AG5284 PJ5:1A1:mKate2* | This study |
| KW069N | *C. necator AG5284 PJ5:1B1:mKate2* | This study |
| KW070N | *C. necator AG5284 PJ5:1C1:mKate2* | This study |
| KW071N | *C. necator AG5284 PJ5:1D1:mKate2* | This study |
| KW072N | *C. necator AG5284 PJ5:2A1:mKate2* | This study |
| KW073N | *C. necator AG5284 PJ5:2B1:mKate2* | This study |
| KW074N | *C. necator AG5284 PJ5:2C1:mKate2* | This study |
| KW075N | *C. necator AG5284 PJ5:2D1:mKate2* | This study |
| KW076N | *C. necator AG5284 PJ5:NoCis:mKate2* | This study |

**Supplemental Table 4.** *Average fluorescence values for CRs measured on the on the BD FACSAria III flow cytometer.*

|  | ***P. putida* (LB)** | ***P. putida* (M9)** | ***C. glutamicum* (integrated)** | ***C. glutamicum* (plasmid)** | ***C. necator*** |
| --- | --- | --- | --- | --- | --- |
| **NEG** | 10 ± 1 | 3 ± 1 | 176 ± 46 | 96 ± 3 | 24 ± 15 |
| **1A1** | 51 ± 2 | 27 ± 1 | 367 ± 64 | 794 ± 8 | 161 ± 7 |
| **1B1** | 54 ± 1 | 27 ± 0 | 521 ± 55 | 1377 ± 35 | 192 ± 11 |
| **1C1** | 36 ± 2 | 30 ± 2 | 519 ± 60 | 1488 ± 48 | 727 ± 115 |
| **1D1** | 91 ± 11 | 164 ± 2 | 613 ± 72 | 1824 ± 58 | 821 ± 145 |
| **2A1** | 630 ± 75 | 556 ± 42 | 903 ± 62 | 2045 ± 54 | 333 ± 18 |
| **2B1** | 1888 ± 251 | 2000 ± 140 | 738 ± 52 | N/A | 11620 ± 913 |
| **2C1** | 2602 ± 290 | 1982 ± 135 | 941 ± 56 | 2036 ± 61 | 11488 ± 1383 |
| **2D1** | 2978 ± 86 | 2152 ± 32 | 2155 ± 272 | 5089 ± 87 | 9422 ± 876 |
| **NC** | 24082 ± 2128 | 25053 ± 733 | 12812 ± 855 | 35054 ± 830 | 41894 ± 4244 |

**Supplemental Table 5.** *Average fluorescence values for CRs measured on the Tecan Infinite M200 plate reader.*

|  | ***P. putida* (LB)** | ***P. putida* (M9)** | ***C. glutamicum* (integrated)** | ***C. glutamicum* (plasmid)** | ***C. necator*** |
| --- | --- | --- | --- | --- | --- |
| **NEG** | 5901 ± 617 | 491 ± 47 | 4634 ± 23 | 11845 ± 585 | 1710 ± 228 |
| **1A1** | 7553 ± 890 | 923 ± 23 | 4787 ± 76 | 13643 ± 247 | 2076 ± 68 |
| **1B1** | 7690 ± 207 | 882 ± 131 | 4738 ± 29 | 13900 ± 478 | 1871 ± 152 |
| **1C1** | 6876 ± 301 | 973 ± 49 | 4853 ± 243 | 14679 ± 158 | 2685 ± 201 |
| **1D1** | 6487 ± 139 | 2437 ± 5 | 4762 ± 353 | 15290 ± 940 | 2437 ± 331 |
| **2A1** | 17448 ± 1454 | 8499 ± 157 | 5110 ± 221 | 15877 ± 873 | 1879 ± 128 |
| **2B1** | 33378 ± 1729 | 18691 ± 272 | 4872 ± 42 | N/A | 14516 ± 709 |
| **2C1** | 50078 ± 1250 | 21064 ± 527 | 4824 ± 217 | 15102 ± 413 | 14533 ± 479 |
| **2D1** | 51058 ± 3718 | 27475 ± 1442 | 5754 ± 344 | 18626 ± 127 | 12315 ± 759 |
| **NC** | 350337 ± 22540 | 322283 ± 7129 | 15160 ± 1646 | 50333 ± 2631 | 45859 ± 2447 |

**References**

Elmore, J. R., Dexter, G. N., Baldino, H., Huenemann, J. D., Francis, R., Peabody, G. L., Martinez-Baird, J., Riley, L. A., Simmons, T., Coleman-Derr, D., Guss, A. M., & Egbert, R. G. (2023). High-throughput genetic engineering of nonmodel and undomesticated bacteria via iterative site-specific genome integration. *Science Advances*, *9*(10), eade1285. <https://doi.org/doi:10.1126/sciadv.ade1285>

Pandey, N., Davison, S. A., Krishnamurthy, M., Trettel, D. S., Lo, C. C., Starkenburg, S., Wozniak, K. L., Kern, T. L., Reardon, S. D., Unkefer, C. J., Hennelly, S. P., & Dale, T. (2022). Precise Genomic Riboregulator Control of Metabolic Flux in Microbial Systems. *ACS Synth Biol*. <https://doi.org/10.1021/acssynbio.1c00638>

Velasquez-Guzman, J. C., Huttanus, H. M., Morales, D. P., Werner, T. S., Carroll, A. L., Guss, A. M., Yeager, C. M., Dale, T., & Jha, R. K. (2024). Biosensors for the detection of chorismate and cis,cis-muconic acid in Corynebacterium glutamicum. J Ind Microbiol Biotechnol. <https://doi.org/10.1093/jimb/kuae024>
